# Short- and Mid-Term Surgical Outcomes of Billroth I Versus Billroth II/Roux-en-Y Reconstruction: A Prospective Observational Cohort Study

**DOI:** 10.3390/medicina61111927

**Published:** 2025-10-27

**Authors:** Catalin Dumitru Cosma, Vlad Olimpiu Butiurca, Marian Botoncea, Cosmin Nicolescu, Cristian Russu, Calin Molnar

**Affiliations:** 1 Faculty of Medicine, George Emil Palade University of Medicine, Pharmacy, Sciences and Technology of Târgu Mureș, 540139 Târgu Mureș, Romania; catalin.cosma@umfst.ro (C.D.C.); vlad.butiurca@umfst.ro (V.O.B.); cosmin.nicolescu@umfst.ro (C.N.); cristirussu@yahoo.com (C.R.); calin.molnar@umfst.ro (C.M.); 2 General Surgery Clinic No.1, County Emergency Clinical Hospital of Târgu-Mureș, 540136 Târgu-Mureș, Romania

**Keywords:** Billroth I, Billroth II, Roux-en-Y, distal gastrectomy, gastric cancer, surgical outcomes, complications

## Abstract

*Background and Objectives*: The best method for reconstructing the stomach after distal gastrectomy surgery in gastric cancer patients continues to be a subject of ongoing discussion. The most beneficial surgical option for patients is Billroth I (BI), yet surgeons may perform Billroth II and Roux-en-Y (BII/RY) procedures because they are easier to execute, although their impact on recovery complications and postoperative function remains unclear. This prospective observational cohort study compares the short- and mid-term surgical outcomes between BI and BII/RY reconstructions. *Materials and Methods*: We included 150 patients who received curative intent distal gastrectomy at the General Surgical Clinic of Emergency County Hospital in Târgu Mureș, Romania, between October 2021 and December 2024 (72 BI and 78 BII/RY patients), with a mean age of 61.5 ± 10.8 years (60.7% male). The outcomes included recovery parameters, postoperative complications (Clavien–Dindo), and mid-term functional results (PPI use, Los Angeles classification esophagitis, bile reflux gastritis, Sigstad dumping score). Inverse probability of treatment weighting (IPTW) was applied to adjust for baseline covariates. *Results*: The results indicated that IPTW adjustment did not change the baseline demographics, tumor characteristics, or perioperative factors. The baseline characteristics were comparable between groups (*p* > 0.05). There were no significant differences in time to flatus (*p* = 0.12), oral diet (*p* = 0.70), or hospital stay (*p* = 0.69). Major morbidity (Clavien–Dindo ≥ III) occurred in 12.7% overall (*p* = 0.17), and the 90-day mortality was 5.3% (*p* = 1.00). At 6 months, bile reflux gastritis was more frequent after BII/RY (*p* = 0.16), whereas dumping syndrome occurred more often after BI (*p* = 0.16). *Conclusions*: The short-term surgical results, together with the total postoperative complications, showed no difference between the BI and BII/RY reconstruction methods. The study revealed distinct functional results between the two groups during the mid-term assessment, which demonstrates that surgeons should maintain their practice of choosing reconstruction techniques according to patient-specific requirements.

## 1. Introduction

Gastric cancer continues to be one of the leading global malignancies, with 1 million new cases and 769,000 deaths during 2020, which placed it as the fifth most common cancer and fourth leading cause of cancer-related deaths worldwide [1]. The disease maintains its position as a significant health burden in East Asian and Eastern European regions, despite ongoing screening progress, multimodal treatment approaches, and perioperative care improvements [2,3]. Surgical resection stands as the primary treatment for curative purposes, and distal gastrectomy serves as the standard surgical method for removing tumors found in the distal stomach [2,3,4]. Globally, approximately 40–50% of newly diagnosed gastric cancer patients are eligible for curative resection, while the remainder present with advanced or metastatic disease at diagnosis, precluding surgical intervention [2,4,5].

The surgical treatment of gastric cancer has received equal emphasis on oncological radicality and functional preservation according to the international guidelines from the past decades [2,3,5,6,7]. The reconstruction method after distal gastrectomy has become a key determinant of both short- and long-term outcomes. The main goal of reconstruction surgery is to achieve food passage through the digestive system, while reducing postoperative complications and minimizing both nutritional deficiencies and reflux problems [4,5,8]. The best method for reconstruction remains unclear because, globally, surgeons and patients have achieved different results in their practices.

The most “physiological” reconstruction method, Billroth I (gastroduodenostomy), maintains the natural food pathway while producing lower bile reflux rates, better nutritional outcomes, and superior long-term life quality [9,10]. This treatment method has certain restrictions when tumors are located in specific areas or if there is duodenal involvement, as well as technical difficulties after extensive lymph node removal.

By contrast, Billroth II and Roux-en-Y reconstructions are widely applied alternatives, particularly when Billroth I is not feasible [10,11]. The Billroth II procedure is easy to perform; however, it leads to higher chances of duodenogastric reflux and remnant gastritis. Roux-en-Y is recommended for minimizing bile reflux and enhancing postoperative function. These techniques result in longer surgical procedures, Roux-en-Y stasis syndrome, and other complications [4,5].

Studies have investigated which reconstruction methods provide the most beneficial advantages. The results of extensive multicenter research in Korea showed that Billroth I and Billroth II surgeries produced different treatment results for patients [10]. The results of nationwide surveys indicated that surgical methods continue to vary across different regions of the country [11]. The selection and success rates of reconstruction methods seem to be influenced by the patient demographics and healthcare systems, according to survival data from Asian and Western European patient groups [9].

Multiple studies using randomized controlled trials and meta-analyses have investigated this matter, with their findings disagreeing about postoperative complications, nutritional outcomes, and life quality [4,5,6,7,8,10,11]. Most of the available data originate from East Asian populations, with relatively few prospective studies from European centers. The research has thoroughly examined perioperative morbidity, while the literature contains limited information about combined short-term and mid-term complications and functional and nutritional results.

Given this ongoing controversy, the selection of the optimal reconstruction after distal gastrectomy remains an unresolved question. Billroth I is often preferred when technically feasible; however, the true advantages of Roux-en-Y over Billroth II or Billroth I are still debated, especially in non-Asian cohorts.

Therefore, this prospective observational cohort study aimed to compare the short- and mid-term surgical outcomes, postoperative complications, and functional parameters between Billroth I and Billroth II/Roux-en-Y reconstruction following distal gastrectomy in gastric cancer patients.

## 2. Materials and Methods

### 2.1. Study Design and Setting

The study took place at the General Surgical Clinic I within the Emergency County Hospital in Târgu Mureș, Romania, between October 2021 and December 2024. The research followed the STROBE (Strengthening the Reporting of Observational Studies in Epidemiology) guidelines for cohort study reporting in its design and process.

### 2.2. Patient Population

**Figure 1:**
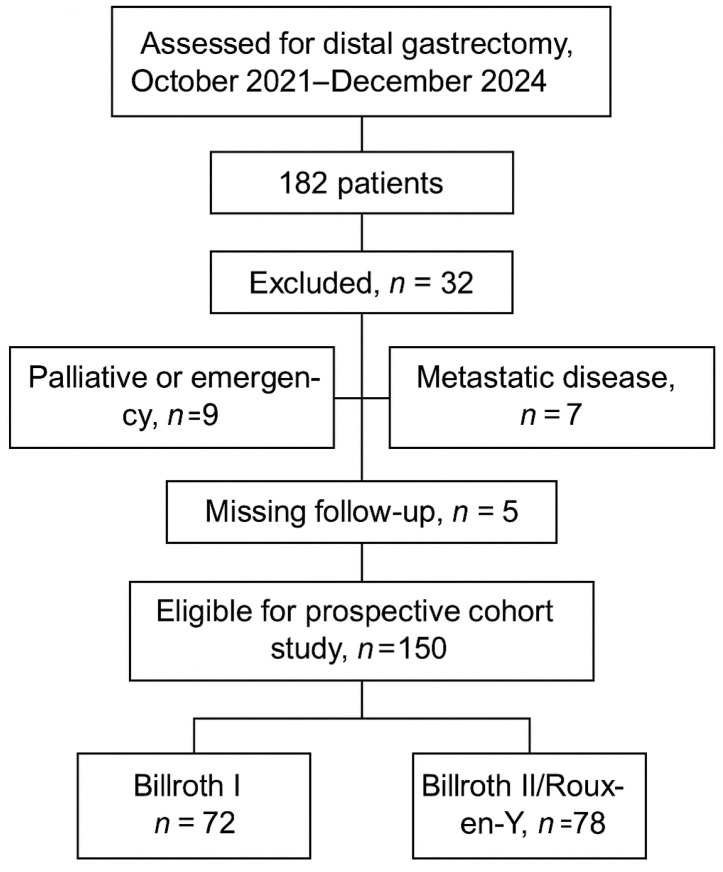
Flowchart of patient selection for inclusion in the prospective cohort study.

Patients were eligible if they were diagnosed with histologically confirmed gastric adenocarcinoma located in the distal stomach or distal gastric body or multicentric tumors confined to the lower body and distal region and underwent curative-intent distal gastrectomy. The study included patients who met three criteria: being at least 18 years old, having tumors located in the distal or lower gastric region, and being able to undergo elective surgery for curative purposes. The study excluded patients who needed total gastrectomy for proximal gastric or esophagogastric junction tumors, those with distant metastases, patients who underwent palliative or emergency procedures, and patients with missing follow-up information. From 182 patients initially assessed for distal gastrectomy (October 2021–December 2024), 32 were excluded (9 palliative/emergency cases, 11 total gastrectomy, 7 metastatic disease, 5 missing follow-up), leaving 150 eligible participants (72 Billroth I and 78 Billroth II/Roux-en-Y) [Figure 1].

The reconstruction decision occurred during surgery based on three main factors, which included the oncological needs, the anatomical possibilities, and the surgeon’s personal choices. Preservation of the right gastroepiploic and right gastric arterial arcades favored selection of Billroth I reconstruction when feasible, whereas high ligation or compromised vascular pedicles prompted Billroth II or Roux-en-Y reconstruction. Billroth I (gastroduodenostomy) was performed when adequate resection margins could be achieved, and sufficient duodenal mobility allowed the creation of a safe tension-free anastomosis. The surgical team chose Billroth II or Roux-en-Y (gastrojejunostomy) when a more proximal or extensive gastric resection was required to ensure negative margins or when duodenal involvement limited mobility. The two reconstruction methods follow international gastric cancer surgery guidelines [2,3] as standard recommendations for clinical practice. The researchers used the inverse probability of treatment weighting (IPTW) to reduce allocation bias in their non-randomized study by making the groups statistically equal through their baseline characteristics.

### 2.3. Surgical Technique

All patients underwent subtotal distal gastrectomy with curative intent, performed according to the oncological principles of gastric cancer surgery. The extent of lymphadenectomy was classified as D1+ or D2, following the Japanese Gastric Cancer Association (JGCA) guidelines [2]. The choice of surgical approach was determined by the operating surgeon and institutional practice standards. Reconstruction methods were applied as follows:

- The Billroth I surgical procedure involved gastroduodenostomy through end-to-end or end-to-side anastomosis between the stomach remnant and duodenum when a safe tension-free anastomosis could be achieved.
- The Billroth II (gastrojejunostomy) procedure with Braun enteroenterostomy was an option, based on surgeon preference, for patients who needed a longer resection margin or had limited duodenal mobility.
- The Roux-en-Y procedure (gastrojejunostomy with Roux limb) served as an antecolic Roux-en-Y gastrojejunostomy with standardized limb length for patients who needed an alternative method to minimize bile reflux and achieve secure tension-free reconstruction.

Anastomoses were primarily hand-sewn using a two-layer technique (inner continuous 3–0 PDS, outer interrupted 3–0 silk). Stapled end-to-side anastomoses (Covidien GIA 60 mm linear stapler) were used selectively when duodenal mobility allowed a tension-free alignment. All stapled lines were reinforced with interrupted seromuscular sutures to ensure hemostasis and anastomotic integrity.

All operations were performed by the same experienced gastrointestinal surgical team within General Surgery Clinic I, ensuring a uniform technique and perioperative management.

### 2.4. Data Collection and Variables

The patient information included age, sex, BMI, ASA class, comorbidities, weight change during 3–6 months, and CRP levels. Neoadjuvant chemotherapy status (yes/no) was recorded for all patients and included as a baseline covariate in the IPTW adjustment. The variables related to tumors included the location, its size, pathological T and N stage, margin status, and treatment with neoadjuvant therapy. The study included four operative variables: the operative time, intraoperative blood loss, extent of lymphadenectomy, and type of anastomosis. The study evaluated the postoperative results through three recovery metrics and four categories of adverse events and their severity levels according to the Clavien–Dindo classification and three early outcome measures. The assessment of mid-term functional results took place at 3 months and 6 months by evaluating the PPI use, the Los Angeles classification of reflux esophagitis with bile reflux gastritis severity, and the Sigstad score for dumping syndrome evaluation.

### 2.5. Outcome Measures

The main study outcome measured the occurrence of severe postoperative complications, which were defined as Clavien–Dindo grade III or higher complications during the first 90 days after surgery. The research team assessed secondary outcomes through evaluations of the total illness rates and particular postoperative issues, healing indicators, and functional outcomes, which included PPI medication use, esophagitis, bile reflux gastritis, dumping syndrome, and death rates at 30 days and 90 days post-surgery. Short-term outcomes were defined as parameters occurring within 90 days postoperatively, whereas mid-term outcomes were assessed at 3 and 6 months after surgery.

### 2.6. Statistical Analysis

The research used means and standard deviations (SD) to present continuous data for normally distributed variables and medians with interquartile ranges (IQR) for variables that did not follow a normal distribution. The researchers conducted independent-sample t-tests or Mann–Whitney U tests for group comparison analysis. Categorical variables were expressed as numbers and percentages and were compared using χ^2^ or Fisher’s exact tests. The study employed the inverse probability of treatment weighting (IPTW) to address confounding through propensity score estimation based on the baseline covariates. The researchers employed standardized mean differences (SMDs) to evaluate the covariate balance at two time points: before and after adjustment. The researchers applied logistic regression for their binary outcome analysis and linear or mixed-effects models for their continuous and time-dependent data. The studies reported their effect sizes through odds ratios (ORs), mean differences, and regression coefficients with 95% confidence intervals (CIs). All analyses were performed using EasyMedStat (SAS, https://www.easymedstat.com/, last accessed 18 October 2025, Paris, France) software. Statistical significance was set at *p* < 0.05 (two-tailed).

## 3. Results

### 3.1. Patient Characteristics

**Figure 2:**
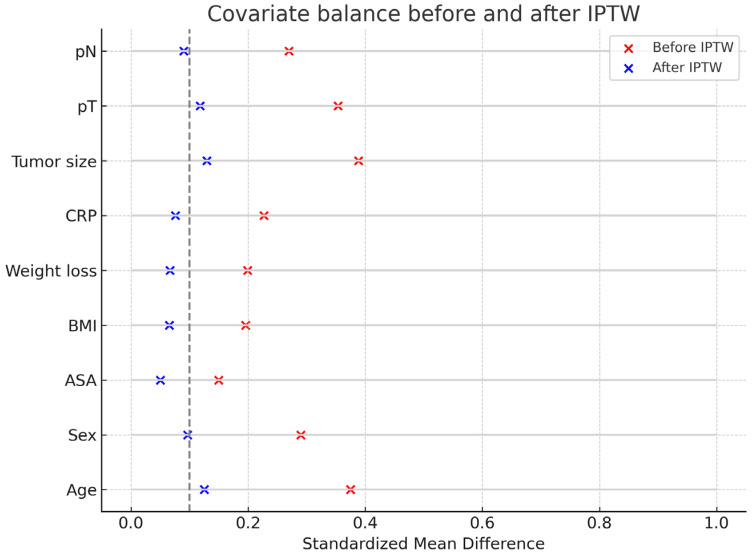
Covariate balance before and after IPTW. The vertical dotted line indicates the standardized mean difference threshold of 0.1, representing acceptable covariate balance between groups.

A total of 150 patients were included, with 72 undergoing Billroth I and 78 undergoing Billroth II/Roux-en-Y reconstruction. The mean age of the cohort was 61.5 ± 10.8 years, with a male predominance of 60.7%. No significant differences were observed between groups with respect to ASA class distribution, BMI, weight loss, CRP, tumor size, pT or pN stage, or neoadjuvant therapy. The R1 resection rate was 9.3% overall, with a slightly higher frequency in Billroth II/Roux-en-Y. The covariate balance was confirmed following IPTW adjustment (Table 1, Figure 2).

### 3.2. Short-Term Surgical Outcomes

**Figure 3:**
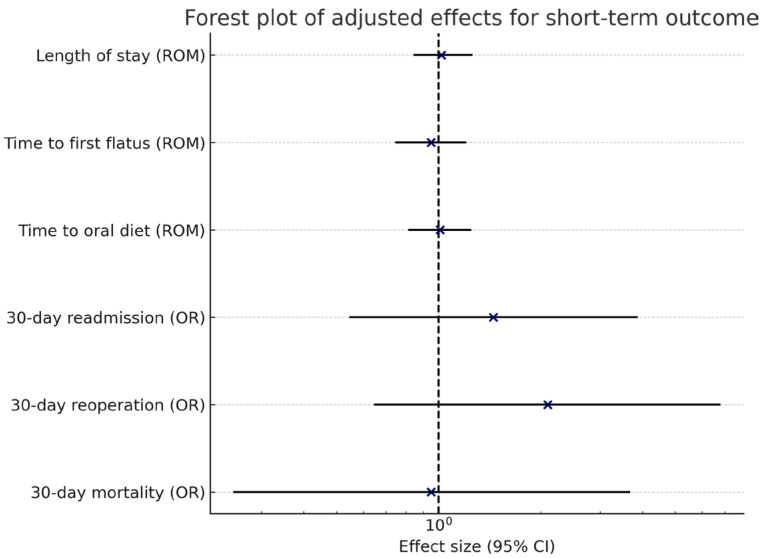
Forest plot of adjusted effects for short-term outcomes. Blue crosses indicate effect estimates, solid lines show 95% confidence intervals, and the dotted line represents the line of no effect (1.0).

**Figure 4:**
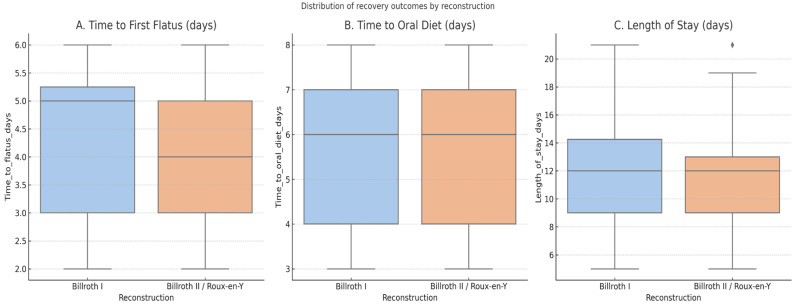
Distribution of postoperative recovery outcomes (time to first flatus, time to oral diet, and hospital stay) by reconstruction type (Billroth I vs. Billroth II/Roux-en-Y). The dot represents outlier values beyond the interquartile range.

The median time to first flatus was 4 days, time to oral diet 6 days, and hospital stay 12 days. There were no significant differences between the reconstruction groups in any of these recovery parameters. Thirty-day readmission occurred in 11.3% of patients, reoperation in 5.3%, and 30-day mortality in 4.0%. The ninety-day mortality was 5.3% overall. Adjusted analyses did not demonstrate significant treatment-related effects across recovery endpoints or adverse events (Table 2, Figure 3 and Figure 4).

### 3.3. Mid-Term Functional Outcomes

**Figure 5:**
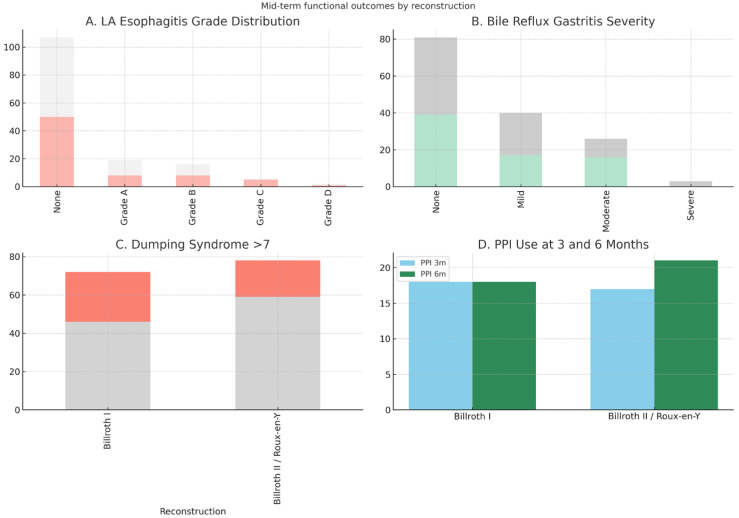
Comparison of mid-term functional outcomes (PPI use, Los Angeles classification of esophagitis, bile reflux gastritis severity, and Sigstad dumping score) between Billroth I and Billroth II/Roux-en-Y groups. Blue and green bars represent the Billroth I group, while orange and gray bars represent the Billroth II/Roux-en-Y group.

At 3 and 6 months, approximately one-quarter of patients required PPI therapy. LA esophagitis was identified in 28.7% overall, predominantly Grades A and B, with no significant intergroup differences. Bile reflux gastritis was more frequent after Billroth II/Roux-en-Y, including severe forms in 3.8% of cases. Dumping syndrome, defined as a Sigstad score >7, occurred in 30.0% of patients, with a higher prevalence in Billroth I (36.1% vs. 24.4%) (Table 3, Figure 5).

### 3.4. Postoperative Complications

The overall morbidity was 32.7%. The most common events were wound infection (7.3%), pulmonary complications (8.7%), and intra-abdominal abscess (4.7%). Duodenal stump leak was observed exclusively in the Billroth II/Roux-en-Y group. Reoperation was required in 5.3% of patients. The complication severity according to the Clavien–Dindo classification was comparable between groups, with 19.3% experiencing Grade II, 12.7% Grade III, and 8.7% Grade V events (Table 4 and Table 5). The slight difference between mortality figures reported in Table 2 and Table 5 arises from one late postoperative death (>90 days) that was included under Grade V in the Clavien–Dindo classification for completeness, but was not counted in the 90-day mortality rate used for short-term outcomes.

## 4. Discussion

This prospective observational cohort study compared short- and mid-term surgical outcomes between Billroth I and Billroth II/Roux-en-Y reconstruction following distal gastrectomy for gastric cancer. The study produced four essential results: (i) The three reconstruction methods showed no significant differences in postoperative recovery times for flatus production, oral diet return, and hospital stay duration. (ii) The rates of major postoperative complications and 30-/90-day mortality showed no significant differences between the reconstruction groups after risk factor adjustment. (iii) The two reconstruction methods produced different functional results during the mid-term period, as the Billroth II/Roux-en-Y patients experienced more bile reflux gastritis, while the Billroth I patients developed dumping syndrome more frequently. (iv) The study found that the reflux esophagitis rates (Los Angeles classification) were equivalent between all treatment groups.

The research findings confirm previous studies showing that Billroth I reconstruction leads to better digestive passage, increasing the chance of dumping syndrome, while Billroth II and Roux-en-Y procedures minimize the duodenogastric reflux, which creates problems with bile stasis and Roux-related complications [12,13,14,15,16,17,18,19,20,21,22,23,24,25,26,27,28,29,30,31]. In a large multicenter Korean analysis, Kang et al. demonstrated differences in perioperative morbidity between Billroth I and Billroth II [10], findings echoed in our cohort, although our IPTW-adjusted analysis suggested no significant early advantage for either method. The South Korean nationwide survey data revealed reconstruction methods followed distinct patterns, because surgeons selected different techniques, and patients had different characteristics [11].

Several randomized controlled trials have directly compared Billroth I and Roux-en-Y. Kimura et al. found no survival difference at 5 years, but the nutritional recovery was superior after Billroth I [18]. Nakamura et al. and Takiguchi et al. demonstrated that the long-term quality of life and functional outcomes were better after Billroth I, though Roux-en-Y reduced bile reflux [23,29]. This study’s results supported these patterns, because nutritional recovery (indirectly assessed through early recovery) showed no substantial differences; however, Billroth II/Roux-en-Y patients developed bile reflux gastritis more often. This is consistent with the findings of Wu et al. [31] and Okuno et al. [32], who stressed that bile reflux develops as a chronic condition after gastrojejunostomy reconstruction surgeries.

Multiple studies have tried to answer this question through meta-analyses. Zong et al. [13] and Xiong et al. [14] reported that Roux-en-Y reconstruction reduced reflux symptoms but at the cost of increased operative complexity. The Cochrane review [19] showed no survival benefit and no major complication variations between the two procedures, while it detected functional outcome variations in terms of bile reflux and dumping symptoms. This study contributes European data to the Asian-dominant research field, which supports the practice of tailoring reconstruction methods to each patient rather than following a single standard approach.

Interestingly, we observed a higher proportion of dumping syndrome in the Billroth I group. This observation is in agreement with Yang et al. [33] and Hirao et al. [24], who reported more frequent postprandial symptoms in Billroth I reconstructions. The direct flow of chyme from the stomach to the duodenum following Billroth I surgery leads to faster intestinal movement, which increases the risk of dumping. Conversely, bile reflux was more common in Billroth II/Roux-en-Y, which reflects the altered bile flow dynamics and has been extensively reported in both Asian RCTs [18,23,25] and Western cohorts [26].

The study benefited from its prospective design, predefined endpoints, and statistical adjustment through IPTW, which reduces bias from non-random reconstruction method distribution. The study provided a complete evaluation through its assessment of short-term surgical complications together with mid-term functional and endoscopic results, which exceeded the scope of many previous retrospective studies.

Nonetheless, some limitations must be acknowledged. The study was conducted at a single location, which restricts the ability to apply its findings to other settings. The research study monitored participants for six months to assess the functional results; however, future studies need to follow participants for longer periods to determine the nutritional effects, oncologic outcomes, and survival rates. The IPTW adjustment method decreased the baseline imbalances; however, non-randomized studies face ongoing difficulties in removing all residual confounding effects. The study had a limited number of patients who received endoscopic evaluation between 3 and 6 months, which could have resulted in underdiagnosis of reflux-related pathologies.

Our research confirms that different reconstruction methods have their own strengths and weaknesses, which prevent any single method from being the best for all applications. Billroth I remains preferable when technically feasible, given its physiological passage and lower bile reflux risk, but surgeons must weigh this against the higher risk of dumping syndrome. The Billroth II/Roux-en-Y procedures should be used for patients who need extensive resection areas or have restricted duodenal movement. Future studies need to conduct multicenter European randomized controlled trials (RCTs) that follow patients for extended periods to evaluate the nutritional results, oncologic outcomes, functional recovery, and patient-reported results for determining the best reconstruction method in gastric cancer surgery.

## 5. Conclusions

In this prospective cohort study, the short-term surgical outcomes and major postoperative morbidity did not differ significantly between Billroth I and Billroth II/Roux-en-Y reconstructions after distal gastrectomy for gastric cancer. The two surgical approaches produced different functional results during the mid-term period, as Billroth I patients developed dumping syndrome more often than Billroth II/Roux-en-Y patients, who, in turn, experienced more bile reflux gastritis. The research demonstrates that both methods continue to be effective while following the established guidelines, because surgeons select reconstruction techniques based on the surgical conditions and individual patient needs.

## Figures and Tables

**Table 1:** Baseline and perioperative characteristics of patients undergoing Billroth I versus Billroth II/Roux-en-Y reconstruction.

Variable	Billroth I (*n* = 72)	Billroth II/RY (*n* = 78)	Total (*n* = 150)	*p*-Value	SMD
Demographics					
Age, years (mean ± SD; median [IQR])	60.4 ± 10.6; 60.0 [51.8–71.0]	62.5 ± 11.0; 64.5 [53.0–71.0]	61.5 ± 10.8; 61.0 [52.0–71.0]	0.21	0.19
Sex, *n* (%)	Male 42 (58.3) Female 30 (41.7)	Male 49 (62.8) Female 29 (37.2)	Male 91 (60.7) Female 59 (39.3)	0.59	0.09
General status					
ASA class, *n* (%)	I: 9 (12.5) II: 35 (48.6) III: 19 (26.4) IV: 9 (12.5)	I: 9 (11.5) II: 38 (48.7) III: 26 (33.3) IV: 5 (6.4)	I: 18 (12.0) II: 73 (48.7) III: 45 (30.0) IV: 14 (9.3)	0.54	0.14
BMI (kg/m^2^)	23.8 ± 3.3; 24.3 [20.8–26.0]	23.6 ± 3.6; 23.8 [20.9–25.9]	23.7 ± 3.5; 24.1 [20.8–25.9]	0.72	0.05
Weight loss 3–6 m (%)	10.4 ± 6.1; 11.2 [5.6–16.1]	10.2 ± 6.3; 10.8 [4.8–15.4]	10.3 ± 6.2; 10.9 [5.6–15.7]	0.82	0.03
CRP (mg/L)	25.8 ± 19.5; 24.7 [7.9–39.2]	22.9 ± 18.7; 20.4 [7.7–35.6]	24.2 ± 19.1; 22.9 [7.7–37.1]	0.34	0.16
Tumor characteristics					
Tumor site, *n* (%)	Distal: 44 (61.1) Body: 19 (26.4) Multicentric: 9 (12.5)	Distal: 50 (64.1) Body: 20 (25.6) Multicentric: 8 (10.3)	Distal: 94 (62.7) Body: 39 (26.0) Multicentric: 17 (11.3)	0.89	0.05
Tumor size (cm)	5.4 ± 1.9; 5.5 [4.5–6.6]	5.6 ± 2.2; 5.4 [4.2–7.2]	5.5 ± 2.1; 5.4 [4.3–6.9]	0.58	0.10
pT stage	T1: 12 (16.7) T2: 24 (33.3) T3: 27 (37.5) T4: 9 (12.5)	T1: 11 (14.1) T2: 27 (34.6) T3: 28 (35.9) T4: 12 (15.4)	T1: 23 (15.3) T2: 51 (34.0) T3: 55 (36.7) T4: 21 (14.0)	0.91	0.07
pN stage	N0: 20 (27.8) N1: 22 (30.6) N2: 21 (29.2) N3: 9 (12.5)	N0: 18 (23.1) N1: 25 (32.1) N2: 22 (28.2) N3: 13 (16.7)	N0: 38 (25.3) N1: 47 (31.3) N2: 43 (28.7) N3: 22 (14.7)	0.74	0.09
Margin status	R0: 66 (91.7) R1: 6 (8.3)	R0: 70 (89.7) R1: 8 (10.3)	R0: 136 (90.7) R1: 14 (9.3)	0.69	0.06
Neoadjuvant therapy	Yes: 24 (33.3) No: 48 (66.7)	Yes: 28 (35.9) No: 50 (64.1)	Yes: 52 (34.7) No: 98 (65.3)	0.75	0.05
Surgical factors					
Lymphadenectomy (D1+/D2)	D1+: 20 (27.8) D2: 52 (72.2)	D1+: 24 (30.8) D2: 54 (69.2)	D1+: 44 (29.3) D2: 106 (70.7)	0.68	0.07
Operative time (min)	201 ± 38; 198 [173–224]	205 ± 41; 201 [179–229]	203 ± 40; 199 [176–226]	0.49	0.10
Blood loss (mL)	289 ± 65; 286 [240–330]	295 ± 71; 292 [246–342]	292 ± 68; 290 [242–336]	0.57	0.09

Continuous variables: independent samples *t*-test (if normally distributed by Shapiro–Wilk) or Mann–Whitney U test. Categorical variables: χ^2^ test or Fisher’s exact test when expected counts <5. Effect size: Standardized mean difference (SMD) reported.

**Table 2:** Short-term surgical outcomes of patients undergoing Billroth I versus Billroth II/Roux-en-Y reconstruction.

Outcome	Billroth I (*n* = 72)	Billroth II/RY (*n* = 78)	Total (*n* = 150)	*p*-Value	SMD
Time to first flatus (days)	4.3 ± 1.4; 5.0 [3.0–5.2]	3.9 ± 1.4; 4.0 [3.0–5.0]	4.1 ± 1.4; 4.0 [3.0–5.0]	0.12	0.25
Time to oral diet (days)	5.7 ± 1.8; 6.0 [4.0–7.0]	5.8 ± 1.7; 6.0 [4.0–7.0]	5.7 ± 1.7; 6.0 [4.0–7.0]	0.70	−0.07
Length of stay (days)	11.8 ± 3.8; 12.0 [9.0–14.2]	11.6 ± 3.4; 12.0 [9.0–13.0]	11.7 ± 3.6; 12.0 [9.0–14.0]	0.69	0.06
30-day readmission	6 (8.3%)	11 (14.1%)	17 (11.3%)	0.39	−0.18
30-day reoperation	2 (2.8%)	6 (7.7%)	8 (5.3%)	0.33	−0.22
30-day mortality	3 (4.2%)	3 (3.8%)	6 (4.0%)	1.00	0.02
90-day mortality	4 (5.6%)	4 (5.1%)	8 (5.3%)	1.00	0.02
Any complication	37 (51.4%)	35 (44.9%)	72 (48.0%)	0.53	0.13

Continuous outcomes (flatus, oral diet, LOS): *t*-test or Mann–Whitney U test. Binary outcomes (readmission, reoperation, mortality, complications): χ^2^ test or Fisher’s exact test. SMD included.

**Table 3:** Mid-term functional outcomes of patients undergoing Billroth I versus Billroth II/Roux-en-Y reconstruction.

Outcome	Billroth I (*n* = 72)	Billroth II/RY (*n* = 78)	Total (*n* = 150)	*p*-Value	SMD
PPI use at 3 months	18 (25.0%)	17 (21.8%)	35 (23.3%)	0.79	0.08
PPI use at 6 months	18 (25.0%)	21 (26.9%)	39 (26.0%)	0.93	−0.04
LA esophagitis	None: 50 (69.4); A: 8 (11.1); B: 8 (11.1); C: 5 (6.9); D: 1 (1.4)	None: 57 (73.1); A: 11 (14.1); B: 8 (10.3); C: 1 (1.3); D: 1 (1.3)	-	0.50	–
Bile reflux gastritis	None: 39 (54.2); Mild: 17 (23.6); Moderate: 16 (22.2)	None: 42 (53.8); Mild: 23 (29.5); Moderate: 10 (12.8); Severe: 3 (3.8)	-	0.16	–
Dumping score (Sigstad)	4.9 ± 4.9; 5.0 [1.6–8.8]	4.4 ± 4.5; 4.8 [1.1–7.0]	4.6 ± 4.7; 4.9 [1.2–7.5]	0.40	0.12
Dumping syndrome >7	26 (36.1%)	19 (24.4%)	45 (30.0%)	0.16	0.26

Continuous (Dumping score): *t*-test or Mann–Whitney U test. Ordinal (LA esophagitis, bile reflux): χ^2^ test for trend or Fisher’s exact test if sparse. Binary (PPI use, Dumping >7): χ^2^ test or Fisher’s exact test.

**Table 4:** Postoperative complications by type in patients undergoing Billroth I versus Billroth II/Roux-en-Y reconstruction.

Complication Type	Billroth I (*n* = 72)	Billroth II/RY (*n* = 78)	Total (*n* = 150)	*p*-Value
Anastomotic leak	2 (2.8%)	3 (3.8%)	5 (3.3%)	1.00
Duodenal stump leak	–	2 (2.6%)	2 (1.3%)	–
Intra-abdominal abscess	3 (4.2%)	4 (5.1%)	7 (4.7%)	0.74
Postoperative bleeding	2 (2.8%)	2 (2.6%)	4 (2.7%)	1.00
Wound infection	5 (6.9%)	6 (7.7%)	11 (7.3%)	0.87
Pulmonary	6 (8.3%)	7 (9.0%)	13 (8.7%)	0.88
Cardiovascular	3 (4.2%)	4 (5.1%)	7 (4.7%)	0.74
Venous thromboembolism	1 (1.4%)	1 (1.3%)	2 (1.3%)	1.00
Stricture	1 (1.4%)	2 (2.6%)	3 (2.0%)	1.00
Reoperation	2 (2.8%)	6 (7.7%)	8 (5.3%)	0.33

All binary categorical: χ^2^ test or Fisher’s exact test. “–” indicates not applicable (e.g., duodenal stump leak in BI). A single patient may present more than one complication type; therefore, the totals may exceed the number of affected patients.

**Table 5:** Postoperative complications classified according to the Clavien–Dindo system.

Clavien–Dindo Grade	Billroth I (*n* = 72)	Billroth II/RY (*n* = 78)	Total (*n* = 150)	*p*-Value
Grade 0 (no complication)	35 (48.6%)	43 (55.1%)	78 (52.0%)	0.47
Grade I (moderate)	5 (6.9%)	4 (5.1%)	9 (6.0%)	0.73
Grade II (minor)	14 (19.4%)	15 (19.2%)	29 (19.3%)	0.97
Grade III (major)	6 (8.3%)	13 (16.7%)	19 (12.7%)	0.17
Grade IV (life-threatening, ICU management)	4 (5.6%)	7 (9.0%)	11 (7.3%)	0.42
Grade V (death)	7 (9.7%)	6 (7.7%)	13 (8.7%)	0.69
Any complication (I–V)	37 (51.4%)	35 (44.9%)	72 (48.0%)	0.53

Each grade was compared between BI and BII/RY using the χ^2^ test or Fisher’s exact test. “Any complication (≥II)” compared with the χ^2^ test. Each patient counted once according to the most severe event. Grades I–V according to Clavien–Dindo classification.

## Data Availability

The data that support the findings of this study are available from the main author, C.C., upon request, catalin.cosma@umfst.ro. Within the limits of the ethical considerations of the hospital commission.

## Abbreviations

The following abbreviations are used in this manuscript:

ASA	American Society of Anesthesiologists
BI	Billroth I
BII/RY	Billroth II/Roux-en-Y
BMI	Body Mass Index
BRG	Bile Reflux Gastritis
CI	Confidence Interval
CONUT	Controlling Nutritional Status
CRP	C-Reactive Protein
IPTW	Inverse Probability of Treatment Weighting
IQR	Interquartile Range
JGCA	Japanese Gastric Cancer Association
LOS	Length of Stay
OR	Odds Ratio
PPI	Proton Pump Inhibitor
QoL	Quality of Life
RCT	Randomized Controlled Trial
SD	Standard Deviation
SMD	Standardized Mean Difference
